# Social network ties before and after retirement: a cohort study

**DOI:** 10.1007/s10433-021-00604-y

**Published:** 2021-03-01

**Authors:** M. Kauppi, M. Virtanen, J. Pentti, V. Aalto, M. Kivimäki, J. Vahtera, S. Stenholm

**Affiliations:** 1 Finnish Institute of Occupational Health, Turku and Helsinki, Finland; 2 School of Educational Sciences and Psychology, University of Eastern Finland, Joensuu, Finland; 3 Clinicum, Faculty of Medicine, University of Helsinki, Helsinki, Finland; 4 Department of Public Health, University of Turku and Turku University Hospital, Turku, Finland; 5 Centre for Population Health Research, University of Turku and Turku University Hospital, Turku, Finland; 6 Department of Epidemiology and Public Health, University College London Medical School, London, UK

**Keywords:** Social relations, Work, Longitudinal study, Cohort study, Retirement

## Abstract

Social networks are associated with individual’s health and well-being. Working life offers opportunities to create and maintain social networks, while retirement may change these networks. This study examined how the number of ties in social network changes across the retirement transition. The study population consisted of 2319 participants (84% women, mean age 63.2 years) from the Finnish Retirement and Aging study. Information about social network ties, including the number of ties in the inner, middle and outer circles of the social convoy model, was gathered using annual postal surveys before and after retirement. Three repeat surveys per participant covered the retirement transition and the post-retirement periods. Mean number of network ties was 21.6 before retirement, of which 5.6 were situated in the inner, 6.9 in the middle and 9.1 in the outer circle. The number of ties in the outer circle decreased by 0.67 (95% CI − 0.92, − 0.42) during the retirement transition period, but not during the post-retirement period (0.11, 95% CI − 0.33, 0.12) (interaction period * time, *p* = 0.006). The pattern of change in these ties did not differ by gender, occupational status, marital status, number of chronic diseases and mental health during the retirement transition period. The number of ties in the inner and middle circles overall did not decrease during these periods. The number of peripheral relationships decreased during the retirement transition but not after that, suggesting that the observed reduction is more likely to be associated with retirement rather than aging.

## Introduction

Social networks may have an important role in health and well-being. Among older adults, larger social networks have been shown to protect from cognitive decline (Crooks et al. 2008) and mortality (Ellwardt et al. 2015; Kauppi et al. 2018). Furthermore, quality of social ties assessed for example by perceived reciprocity (Wahrendorf et al. 2010) and higher level of social engagement have been shown to associate with better mental (Glass et al. 2006; Wahrendorf et al. 2010) and physical health (Golden et al. 2009; Wahrendorf et al. 2010; Thomas 2012).

Working life offers opportunities to create and maintain social networks and adopt important social roles, and after retirement these work-related roles and networks naturally change. On the one hand, contacts with coworkers are apt to diminish markedly along with retirement. On the other hand, retirement increases the available time to spend with family, friends, neighbors and voluntary work settings enabling to strengthen and even create new social ties. In this study we aim to examine the potential changes in social networks along with retirement.

One theory by which the dynamics, both regarding the size and closeness of social network, might be explained is the convoy model of social relations introduced by Antonucci (1986). According to that theory individuals are surrounded by supportive others who move with them throughout the life course. In the social convoy model these personal network relations are indicated by three concentric circles referring to the levels of closeness. These may include diverse relations, such as family members, friends, neighbors and coworkers, and thus represent the whole social network of an individual. According to the social convoy model, these relations are differently affected by changes in a person’s circumstances, so that the closest relations are assumed to be highly stable throughout the life span, while less close relations which are often role-related are less stable and more likely to be affected by life changes, such as retirement (Antonucci and Akiyama 1987).

Previous studies on social networks among older adults have mostly concentrated either on age-related changes, not taking retirement into account (Shaw et al. 2007; Wrzus et al. 2013; Cornwell et al. 2014; Suanet and Huxhold 2018) or have compared older workers with those who are retired (Moen 2000; Fletcher 2014; Wan and Antonucci 2017). To our knowledge, only few previous studies have prospectively assessed the changes in social network ties across the retirement transition and the results have been contradictory. One study, based on a nationally representative stratified random sample of older Americans aged 57–70 years, showed that retirement was associated with a decrease in the number of network ties during a 5-year follow-up (Patacchini and Engelhardt 2016). On the other hand, the study by Sabbath et al. (2015), based on data of French utility workers aged 51–65 years with the mean retirement age of 55 years, showed an increase in the size of familial and friend social network after retirement. Two Dutch studies, based on data of retiring men aged 55–65 (mean retirement age around 61 years) and 54–81 years at baseline, have shown only minor changes in social network size during retirement (van Tilburg 2003, 1992).

In addition, it has been suggested that women, those with a partner, with higher socioeconomic status and better health have generally higher number of social network ties (Patacchini and Engelhardt 2016; Sabbath et al. 2015; van Tilburg and van Groenou 2003), but it is not known whether the potential changes are similar in men and women, those who are married or cohabiting versus not married, and across occupational or health status groups. Some studies have suggested that women’s networks are more likely to shrink after retirement (Patacchini and Engelhardt 2016), while others have shown that women experience improved contact and emotional support (Fischer and Beresford 2015), maintain more friendship ties (Stevens and van Tilburg 2011) and thus increase their social network over time (Schwartz and Litwin 2018). With respect to marital status the changes in social network may differ because in retirement married or cohabiting subjects are likely to focus more on their families and lose strong support relationships with coworkers, while single individuals may have a stronger incentive to stay in contact with former coworkers or replace these ties with other ties in case they are lost during the retirement transition. Previous results concerning socioeconomic status and health suggest low midlife socioeconomic status and poor health to associate with lower social engagement and decrease in the number of social network ties after retirement (Sabbath et al. 2015).

To increase our understanding how retirement associates with changes in social network ties, this study aimed to examine these changes in social network, using the social convoy model, during the transition from work to statutory retirement. Our research questions (RQ) were: (1) How do the number of social ties in total and of different levels of closeness change across the retirement transition? (2) Do the changes in social ties differ by gender, marital status, occupational status and health status?

## Methods

### Study design

The present study is based on the Finnish Retirement and Aging Study (FIREA), an ongoing follow-up study of aging public sector employees in Finland. The study design has been described in detail elsewhere (Leskinen et al. 2018). In brief, the eligible population for the FIREA study included all public sector employees whose estimated individual retirement date was between 2014 and 2019, and who were working in one of the 27 municipalities in Southwest Finland or in the 9 selected cities or 5 hospital districts around Finland in 2012, and thus represented well Finnish public sector employees close to retirement. Information on the estimated individual retirement date from the municipal employer was obtained from the pension insurance institute for the municipal sector in Finland (Keva). According to the public sector Employees’ Pension Act, from 2005 onward public sector employees can retire on a statutory basis after the age of 63 but at the latest at 68. In some occupations the employees may have kept the lower retirement age determined in previous pension act (e.g., 60 years for primary school teachers, 58 for practical nurses). All employees are insured by the employer and are thus entitled to receive earnings-related pension after retirement. Employees may however continue working after their individual pensionable date, which accrue pension income level until the age of 68. In Finland, almost one-fifth (16–20%) of public sector employees were working beyond their individual pensionable date in 2014–2019 (Kauppi et al. 2021).

Participants were first contacted 18 months prior to their estimated retirement date by sending a questionnaire, which was thereafter sent annually, five times in maximum. Actual date of retirement was reported by the participants. Due to the eligibility criteria, large majority of the FIREA participants retired based on their age, not due to work disability. The FIREA study was conducted in line with the Declaration of Helsinki and was approved by the Ethics Committee of Hospital District of Southwest Finland.

**Fig. 1:**
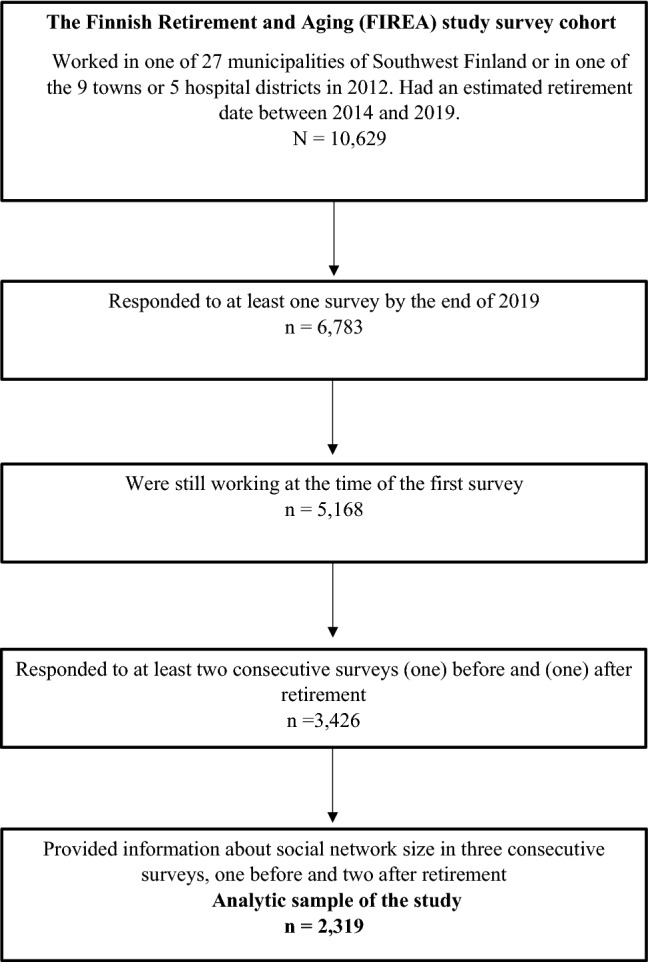
The flowchart of the study

This study was carried out on 2319 respondents of the FIREA study who were working full-time or part-time before retirement and provided valid information on social network size in three consecutive study waves, one before retirement (wave − 1) and two after retirement (waves 1 and 2) (Fig. 1; flowchart of the study).

### Measurement

#### Dependent variable

Social network ties were assessed using a questionnaire, by means of the social convoy model introduced by Antonucci (1986). The model is based on a set of three concentric circles each of which is considered to represent different levels of closeness to the focal person. In the innermost circle, the respondents were asked to indicate with how many people they felt so close that it was hard to imagine life without them (the closest ties). The middle circle referred to those persons who felt not quite that close but still important (close ties) and, in the outer circle, the respondents added the number of those persons who were not already mentioned, but who were close and important enough to belong to the individual’s personal network (less close ties). Total number of network ties was determined by summing up the number of persons in all circles.

#### Covariates

Covariates were chosen on a basis of an a priori assumption of variables associated with number of social network ties (van Tilburg and van Groenou 2003; Ajrouch et al. 2005; Sabbath et al. 2015; Patacchini and Engelhardt 2016). Information about sex, date of birth and occupational status was obtained from the register of the pension insurance institute for the municipal sector in Finland (Keva). Occupational status was categorized into three groups according to the last known occupational title preceding retirement: upper grade non-manual workers (e.g., teachers and physicians), lower grade non-manual workers (e.g., technicians and registered nurses) and manual workers (e.g., cleaners). Information about other covariates was drawn from the survey conducted prior to the retirement (wave − 1). Marital status was categorized into married or cohabiting and not married or other. Number of diagnosed chronic diseases was categorized into 0, 1, ≥ 2 diseases including angina pectoris, myocardial infarction, stroke, claudication, osteoarthritis, osteoporosis, sciatica, fibromyalgia, rheumatoid arthritis, diabetes, migraine and cancer. Mental disorders included diagnosed depression and/or other mental diseases (no, yes).

### Statistical analysis

We used linear regression analyses with generalized estimating equations (GEE), which controls for the intra-individual correlation between repeated measurements using an exchangeable correlation structure and is not sensitive to measurements missing completely at random (Zeger and Liang 1986). To answer our first research question (*How do the number of social ties in total and of different levels of closeness change across the retirement transition?)* we used the GEE modeling to calculate the mean estimates and their 95% confidence intervals (CI) for the total number of network ties and separately for the number of ties in the inner, middle and outer circles at each wave from wave − 1 to wave 2. Then we quantified the changes in the total number of network ties, as well as in the number of ties in each three circles within two time periods: retirement transition period and post-retirement period. The retirement transition period was defined as period from wave − 1 to wave 1 (corresponding approximately 0.5 years before retirement to 0.5 years after retirement) and the post-retirement period as period from wave 1 to wave 2 (corresponding approximately 0.5 to 1.5 years after retirement).

After that we compared the changes between these two periods to examine whether the changes are due to retirement by including period * time interaction term in the GEE model.

Finally, we examined whether the changes during the retirement transition period differed by gender, marital status, occupational status and health status (RQ2). That was done by adding the interaction term subgroup*time to the GEE models. All models were adjusted for age, and sex, as appropriate. All statistical analyses were performed with SAS 9.4 Statistical Package (SAS Institute Inc., Cary, NC).

## Results

Characteristics of the study population before retirement (wave − 1) are shown in Table 1. Mean age of the participants was 63.2 (SD 1.3) years, and 84% were women. Of the participants, 33% had upper grade non-manual occupation and 36% had manual occupation, 73% were married or cohabiting, 25% were free of chronic diseases, and 17% had mental disorders. The final analytical sample was highly similar to the eligible population (n = 10,629) in regard to the background characteristics (mean age 64.1 years, 80% women, 29% in upper grade non-manual occupation and 42% in manual occupation in the eligible population).

**Table 1:** Characteristics of the study population before retirement (wave − 1) (n = 2319)

	Mean	SD
Age in years	63.2	1.3
	*n*	%
Sex		
Men	371	16
Women	1948	84
Occupational status		
Upper grade, non-manual	756	33
Lower grade, non-manual	716	31
Manual	825	36
Marital status		
Married or cohabiting	1650	73
Not married or other	613	27
Number of chronic diseases^a^		
0	571	25
1	818	36
≥ 2	855	38
Mental disorders^b^		
No	1752	83
Yes	360	17

*SD* standard deviation

^a^Chronic diseases include angina pectoris, myocardial infarction, stroke, claudication, osteoarthritis, osteoporosis, sciatica, fibromyalgia, rheumatoid arthritis, diabetes, migraine and cancer

^b^Mental disorders include depression and/or other mental diseases

**Fig. 2:**
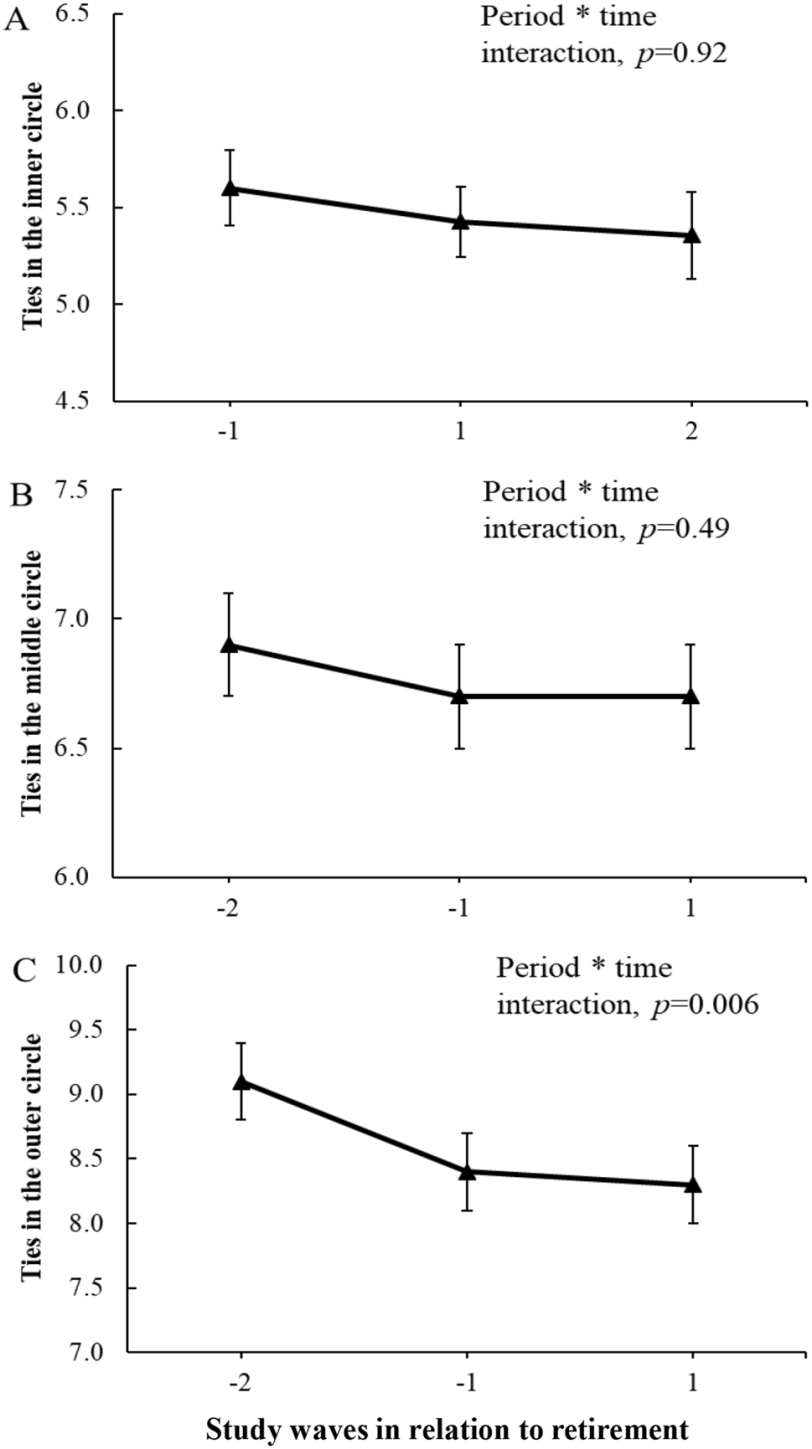
Age- and sex-adjusted mean number of ties in the inner (**a**), middle (**b**) and outer circles (**c**) of social network during the retirement transition period (wave − 1 to wave 1) and the post-retirement period (wave 1 to wave 2) and difference in change between the periods (period * time interaction)

Mean of total number of network ties was 21.6 before retirement, and the number of ties decreased approximately by one person during the retirement transition period (− 0.90, 95% CI − 1.31, − 0.50) but not during the post-retirement period (− 0.22, 95% CI − 0.58, 0.15) (RQ1). As shown in Table 2 and Fig. 2 most marked decrease was observed in the number of less close ties, those in the outer circle. Mean number of these ties was 9.1 before retirement, and this number decreased significantly during the retirement transition period (− 0.67, 95% CI − 0.92, − 0.42) but not during the post-retirement period (− 0.11, 95% CI − 0.33, 0.12). Mean number of ties in the inner circle was 5.6 and in the middle circle 6.9 before retirement, and there was no decrease in ties during the retirement transition period in either of these circles (Tables 3 and 4).

**Table 2:** Mean number of ties in the outer circle of the social convoy model at pre-retirement (wave − 1) and its change (95% CI) during the retirement transition period (waves − 1 to 1) by pre-retirement characteristics

	*n*	%	Pre-retirement (wave − 1)		Retirement transition period (waves − 1 to 1)		*p* value for subgroup * time interaction
			Mean	95% CI	Mean change	95% CI	
All	2319	100	9.1	8.7, 9.4	− 0.67	− 0.92, − 0.42	
Sex							0.48
Men	371	16	9.3	8.7, 10.0	− 0.46	− 1.09, 0.16	
Women	1948	84	8.8	8.6, 9.1	− 0.71	− 0.98, − 0.44	
Occupational status							0.38
Upper grade, non-manual	756	33	9.5	9.1, 10.0	− 0.44	− 0.89, 0.02	
Lower grade, non-manual	716	31	8.7	8.3, 9.2	− 0.87	− 1.30, − 0.45	
Manual	825	36	8.7	8.2, 9.2	− 0.72	− 1.13, − 0.30	
Marital status							0.45
Married or cohabiting	1650	73	9.4	9.0, 9.8	− 0.74	− 1.05, − 0.44	
Not married or other	613	27	8.0	7.5, 8.5	− 0.54	− 0.98, − 0.09	
Number of chronic diseases^a^							0.86
0	571	25	8.8	8.2, 9.3	− 0.82	− 1.30, − 0.33	
1	818	36	9.2	8.7, 9.7	− 0.65	− 1.08, − 0.22	
≥ 2	855	38	9.0	8.5, 9.5	− 0.67	− 1.09, − 0.26	
Mental disorders^b^							0.43
No	1752	83	9.1	8.8, 9.5	− 0.77	− 1.06, − 0.48	
Yes	360	17	8.6	7.9, 9.3	− 0.50	− 1.10, 0.10	

Models are based on linear regression analyses with generalized estimating equations (GEE). Models are adjusted for sex and age, as appropriate

^a^Chronic diseases include angina pectoris, myocardial infarction, stroke, claudication, osteoarthritis, osteoporosis, sciatica, fibromyalgia, rheumatoid arthritis, diabetes, migraine and cancer

^b^Mental disorders include depression and/or other mental diseases

**Table 3:** Mean number of ties in the middle circle of the social convoy model at pre-retirement (wave − 1) and its change (95% CI) during the retirement transition period (waves − 1 to 1) by pre-retirement characteristics

	*n*	%	Pre-retirement (wave − 1)		Retirement transition period (waves − 1 to 1)		*p* value for subgroup * time interaction
			Mean	95% CI	Mean change	95% CI	
All	2319	100	6.9	6.6, 7.1	− 0.18	− 0.36, 0.01	
Sex							0.18
Men	371	16	6.6	6.2, 7.1	0.09	− 0.32, 0.51	
Women	1948	84	7.0	6.8, 7.2	− 0.23	− 0.43, − 0.02	
Occupational status							0.79
Upper grade, non-manual	756	33	7.2	6.8, 7.5	− 0.13	− 0.46, 0.19	
Lower grade, non-manual	716	31	6.5	6.1, 6.8	− 0.11	− 0.43, 0.21	
Manual	825	36	6.9	6.5, 7.3	− 0.26	− 0.58, 0.07	
Marital status							0.64
Married or cohabiting	1650	73	7.1	6.8, 7.4	− 0.20	− 0.43, 0.02	
Not married or other	613	27	6.2	5.8, 6.6	− 0.11	− 0.45, 0.24	
Number of chronic diseases^a^							0.25
0	571	25	6.9	6.5, 7.3	− 0.41	− 0.80, − 0.01	
1	818	36	6.9	6.6, 7.3	0.01	− 0.29, 0.32	
≥ 2	855	38	6.7	6.4, 7.1	− 0.19	− 0.49, 0.11	
Mental disorders^b^							0.63
No	1752	83	6.9	6.6, 7.2	− 0.14	− 0.36, 0.08	
Yes	360	17	6.7	6.2, 7.2	− 0.26	− 0.70, 0.18	

Models are based on linear regression analyses with generalized estimating equations (GEE). Models are adjusted for sex and age, as appropriate

^a^Chronic diseases include angina pectoris, myocardial infarction, stroke, claudication, osteoarthritis, osteoporosis, sciatica, fibromyalgia, rheumatoid arthritis, diabetes, migraine and cancer

^b^Mental disorders include depression and/or other mental diseases

**Table 4:** Mean number of ties in the inner circle of the social convoy model at pre-retirement (wave − 1) and its change (95% CI) during the retirement transition period (waves − 1 to 1) by pre-retirement characteristics

	*n*	%	Pre-retirement (wave − 1)		Retirement transition period (waves − 1 to 1)		*p* value for subgroup * time interaction
			Mean	95% CI	Mean change	95% CI	
All	2319	100	5.6	5.4, 5.8	− 0.06	− 0.20, 0.09	
Sex							0.20
Men	371	16	5.4	5.0, 5.8	− 0.28	− 0.65, 0.09	
Women	1948	84	6.0	5.8, 6.2	− 0.01	− 0.17, 0.14	
Occupational status							0.04
Upper grade, non-manual	756	33	5.6	5.3, 5.9	− 0.29	− 0.53, − 0.04	
Lower grade, non-manual	716	31	5.3	5.0, 5.6	0.17	− 0.09, 0.42	
Manual	825	36	6.0	5.7, 6.3	− 0.05	− 0.31, 0.22	
Marital status							0.002
Married or cohabiting	1650	73	5.8	5.6, 6.0	− 0.21	− 0.39, − 0.03	
Not married or other	613	27	5.0	4.7, 5.3	0.28	0.02, 0.53	
Number of chronic diseases^a^							0.84
0	571	25	5.7	5.3, 6.0	− 0.10	− 0.43, 0.23	
1	818	36	5.5	5.2, 5.8	− 0.13	− 0.37, 0.11	
≥ 2	855	38	5.6	5.3, 5.9	− 0.04	− 0.26, 0.19	
Mental disorders^b^							0.02
No	1752	83	5.7	5.4, 5.9	− 0.16	− 0.33, 0.01	
Yes	360	17	5.2	4.8, 5.7	0.30	− 0.05, 0.66	

Models are based on linear regression analyses with generalized estimating equations (GEE). Models are adjusted for sex and age, as appropriate

^a^Chronic diseases include angina pectoris, myocardial infarction, stroke, claudication, osteoarthritis, osteoporosis, sciatica, fibromyalgia, rheumatoid arthritis, diabetes, migraine and cancer

^b^Mental disorders include depression and/or other mental diseases

We assessed the differences in changes between the retirement transition period and post-retirement period by including period * time interaction term in the models. The results of these models showed that there was a significant difference in change between these periods for the total number of network ties (*p* = 0.04) and for ties in the outer circle (*p* = 0.006), but not for ties in the inner (p = 0.92) or middle circles (*p* = 0.49) (Fig. 2).

When assessing the number of social ties and their changes during the retirement transition period in subgroups (RQ2) we found that before retirement (at wave − 1) the mean number of ties was higher among married or cohabiting persons compared to those not married in the outer (9.4 vs. 8.0) and middle (7.1 vs. 6.2) circles (Tables 2 and 3). During the retirement transition period the number of ties in the outer circle decreased among women (− 0.71, 95% CI − 0.98, − 0.44), those in lower grade non-manual occupations (− 0.87, 95% CI − 1.30, − 0.45) or in manual occupations (− 0.72, 95% CI − 1.13, − 0.30), among married or cohabiting persons (− 0.74, 95% CI − 1.05, − 0.44) as well as among those not married (− 0.54, 95% CI − 0.98, − 0.09), among those with no mental disorders (− 0.77, 95% CI − 1.06, − 0.48) and among all regardless of number of chronic diseases (− 0.82, 95% CI − 1.30, − 0.33 for no chronic diseases, − 0.65, 95% CI − 1.08, − 0.22 for one chronic disease and − 0.67, 95% CI − 1.09, − 0.26 for ≥ 2 chronic diseases). However, no significant difference in change was observed within any of these subgroups (subgroup * time interaction *p* > 0.1). Only a slight decrease was noticed in the number of ties in the middle circle among women and those with no chronic diseases but again no difference of change within any of the subgroups (subgroup * time interaction *p* > 0.1) (Table 3).

As shown in Table 4, before retirement (at wave − 1) women had slightly more ties in the inner circle compared to men (6.0 vs. 5.4), as did those in manual occupations (6.0) compared to those in lower grade non-manual occupations (5.3), and married or cohabiting persons compared to those not married (5.8 vs. 5.0). The pattern of change in these closest ties differed (subgroup * time interaction) according to occupational status (*p* = 0.04), marital status (*p* = 0.002) and mental disorders (*p* = 0.02). The mean number of the closest ties decreased during the retirement transition period among those in upper grade non-manual occupations (− 0.29, 95% CI − 0.53, − 0.04), but not among those with lower level of occupational status (0.17, 95% CI − 0.09, 0.42 for lower grade non-manual and − 0.05, 95% CI − 0.31, 0.22 for manual occupations) and among those who were married or cohabiting (− 0.21, 95% CI − 0.39, − 0.03) but not among single persons (0.28, 95% CI 0.02, 0.53). The decrease in these ties was marginal among those who had no mental disorders (− 0.16, 95% CI − 0.33, 0.01), and no decrease was noticed among those with mental disorders (0.30, 95% CI − 0.05, 0.66).

## Discussion

In this longitudinal study of retiring public sector employees in Finland, we observed a loss of, on average, one tie in personal social network during the retirement transition period but no corresponding change after retirement. The decrease was most marked in less close ties, those in the outer circle. No corresponding change was noticed in closer ties, those in the middle and inner circles. Our results are thus consistent with the previous studies suggesting the less close relationships, for example work-related network ties (van Tilburg 1992, 2003) to decrease over time and closer relationships to be more enduring (Shaw et al. 2007; Suanet et al. 2013; Wrzus et al. 2013). Our findings are also in accordance with the social convoy theory (Antonucci and Akiyama 1987) which assumes that a decrease in the number of social network ties is more likely seen in more peripheral ties than in the closest ties. In addition, the fact that this change was apparent during the retirement transition period but not during the post-retirement period suggests that a decrease in social network ties is due to retirement, thus supporting the assumptions of the social convoy model that the relations are affected by changes in a person’s circumstances.

The decrease in the number of ties in the middle and outer circles was similar during the retirement transition period in all subgroups stratified by sex, occupational status, marital status and health, suggesting that our finding is robust. The number of ties in the inner circle practically did not change during the retirement transition period or the post-retirement period in the total study population. However, during the retirement transition period there was a decrease in these closest ties among those in upper grade non-manual occupations but not among those in lower grade non-manual or manual occupations. Those in upper grade non-manual occupations may be more work-oriented than those with lower occupational status. It may be that they have larger share of coworkers in their closest ties, and thus retirement decreases the number of these ties among them.

A decrease in the closest ties was also noticed among married or cohabiting subjects but not among those who were not married. This may be related to the loss of a spouse/partner due to death or divorce, both events a person living alone cannot encounter (Wrzus et al. 2013). Also, it might be that married or cohabiting subjects are more likely to focus on their families after retirement and thus lose even the closest relationships with former coworkers, while single individuals may have a stronger incentive to stay in contact with their former coworkers or replace these ties with other close ties.

The mean number of the closest ties decreased marginally among those who had no mental disorders, while among those with mental disorders it rather appeared to increase. We have no conclusive explanation to this difference. However, since information about mental disorders was assessed before retirement (at wave − 1), it may have changed during the follow-up potentially affecting the results.

Our findings on decreasing social ties differ from the results of Sabbath et al. (2015) who found in their longitudinal study on French utility workers an overall increase in the size of close social network with family and friends across the retirement. The differences in these findings may be partly related to the operationalization of social network ties. In the study of Sabbath et al. (2015) the size of close family and friend social network was assessed, while we used categorization based on the social convoy model including three concentric circles representing different levels of closeness, potentially including also other types of close relationships. In addition, there was a marked age and gender difference between the studies. In the study of Sabbath et al. (2015) the mean retirement age was around 55 years and 81% of the study sample comprised of men, while in our study the mean retirement age was over 63 years and most participants were women (84%). Therefore, the difference in results may at least partly reflect potential age and gender differences in social network changes across the retirement transition.

It is of note that the time frame used in our study was relatively short, from around 0.5 years before retirement till around 1.5 years after retirement. As retirement may be regarded as a process during which the contacts with former coworkers may be lost first and then compensated by other ties later, the timing of the measurements around retirement may affect the noticeable changes (Moen 2000). This may also explain the different results between our study and the study of Sabbath et al (2015). In their study the assessment points were 13 years apart from each other, and therefore, the timing of retirement relative to pre- and post-retirement measurements has differed considerably from our study. It is thus possible that participants of these studies have been at different stages of retirement process. This idea was supported by the fact that those participants in the study of Sabbath et al. (2015) who had been retired longer were more likely to increase the number of network ties compared to those who had retired more recently.

To our knowledge, this study is one of the first attempts to prospectively examine the changes in individuals’ social networks across the retirement. The main strength of the study is therefore study design allowing an assessment of annual changes in ties of different levels of closeness using repeat data on social network ties before and after retirement. It is important to assess ties with different levels of closeness separately since as it has been shown, less close ties may be particularly beneficial for health and well-being of an individual, even irrespective of the number of the closest ties (Sandstrom and Dunn 2014; Kauppi et al. 2018; Pan and Chee 2019). This may at least partly relate to that compared to the closest ties, less close ties are more often voluntary, less time-consuming and intimate, and are thus likely to avoid some negative social influences. Therefore, the changes in ties with different level of closeness may affect differently on individual’s well-being. Another strength was the homogeneity of our sample which did not include early retirement due to work disability. Thus, there were no major differences in health-related selection around the retirement transition between the participants.

However, some limitations are noteworthy. First, we had no information about the members or quality of relations in personal social network. Yet, as it has been shown previously, the closest ties most often include family members, while less close ties are likely to include for example less close family members and friends (Antonucci et al. 2004). Also, the size of social network, especially the number of the closest ties, can be regarded as an indicator of potentially available support even if we had no information about the quality of relations or actual support provided through them. Second, we could not assess the degree of potential turnover within the social networks. For example, the number of ties in the innermost circles appeared to decrease only slightly across the retirement transition. However, we did not know whether the ties in different circles were the same in each waves of retirement process. It might be that some of the closest ties are being lost and new close ties being achieved as replacements. Also, it might be that some ties which earlier were less close are converted into closer ties during and after retirement. It has been shown that social network turnover may be notable over longer time period, the rate of which is largely affected by some major life events as well as by some background characteristics, such as health and socioeconomic status (Wellman et al. 1997; Cornwell 2015; Badawy et al. 2019). However, since the time frame of the present study was relatively short, it is quite unlikely that the degree of social turnover would be high. Yet, since it has been shown that social network turnover, social disadvantage and poor health may be interlinked (Cornwell 2015), future studies should assess the potential turnover in social networks during the retirement transition and to examine whether the changes in social network characteristics associate, e.g., with an individual’s adaptation to a new life situation. In addition, due to relatively short time period it was not possible to accurately examine potential effects of aging on social network change. However, our finding that the change was apparent during the retirement transition period, and plateaued after that suggest a decrease to be rather due to retirement than to aging.

Our study sample comprised mostly of women which might be a limitation since women are often regarded more gregarious, to have larger and more diverse networks than men and are more likely to participate in the study, which might bias the results. However, this gender distribution represents well public sector employees in Finland (Statistics Finland 2016). Also, our results did not show any differences in changes between the genders over time.

Finally, although our final analytical sample was highly similar to the eligible population in terms of age, gender and occupation, and findings may thus be generalizable to public sector employees in Finland, there might be other, e.g., personality factors, such as loyalty and conscientiousness, which differ between participants and those who did not respond. Future studies should examine whether similar changes in social ties occur in other countries with different work cultures and pension systems.

## Conclusion

In this prospective study with three survey waves around the retirement transition, a decrease in the social network size due to changes in the number of less close ties was observed during the retirement transition period, but not during the post-retirement period. Therefore, the observed reduction is likely to be associated with retirement rather than aging. Further studies with more diverse information on social ties and longer follow-up periods are needed to assess the changes in social networks during and after retirement transition.
